# Inflammatory biomarkers and depression in chronic heart failure: a review of current evidence

**DOI:** 10.1192/j.eurpsy.2026.11032

**Published:** 2026-07-31

**Authors:** A. K. Sikora, S. Fedorov, M. Bibik

**Affiliations:** 1 Ivano-Frankivsk National Medical University, Ivano-Frankivsk, Ukraine; 2 Mazovian Specialist Hospital “Drewnica”, Ząbki, Poland

## Abstract

**Introduction:**

Chronic heart failure (CHF) remains a leading cause of morbidity and mortality worldwide. Depression is a frequent comorbidity in CHF (prevalence ~20-40%) and is linked to worse outcomes including poorer quality of life and increased hospitalizations. Emerging evidence suggests that inflammation and neurohormonal dysregulation may mediate the relationship between CHF and depressive symptoms. Clarifying these links could help in developing more targeted therapeutic strategies.

**Objectives:**

To review recent literature examining associations between inflammatory biomarkers (e.g. TNF-α, interleukins), NT-proBNP, and depressive symptoms, anxiety, or quality of life in patients with CHF.

**Methods:**

A narrative literature search was performed in PubMed, Scopus, and Web of Science for articles published between 2020 and 2025. Criteria: studies in CHF patients; measurement of inflammatory markers or NT-proBNP; assessment of depression, anxiety, or quality of life. Both observational and interventional studies included.

**Results:**

Recent studies confirm that elevated inflammatory markers are associated with depressive symptoms in CHF. For example, in a cohort study, depressive symptomatology correlated positively with NT-proBNP and poorer health status (Balata et al., 2024). TNF-α and IL-6 are frequently reported elevated in CHF and are implicated in myocardial dysfunction and remodeling (Arvunescu et al., 2023). In a study of CHF outpatients, IL-6 levels were significantly correlated with psychosomatic psychopathology and depression indices after controlling for age, gender, NYHA class, etc. However, some studies fail to find a significant association for all cytokines; results are heterogeneous. There is also evidence that NT-proBNP is not only a marker of cardiac stress but is related to psychological distress: higher NT-proBNP levels associated with worse depressive symptoms and lower quality of life (Müller-Tasch et al., 2021).

**Conclusions:**

The weight of recent evidence supports a link between inflammation, elevated NT-proBNP, and depressive symptoms in CHF. Nevertheless, heterogeneity in methods, depression scales, biomarker assays, and patient populations limits firm conclusions. Future longitudinal studies and randomized interventions are required to determine causality and to test whether anti-inflammatory or neurohormonal modulation strategies can improve both mood and cardiac outcomes.

**Disclosure of Interest:**

None Declared

